# My Impression of the Hospital: The Unaided Composition of a Patient Aged Eleven

**Published:** 1913-12-27

**Authors:** 


### HOSPITALS FROM THE PATIENTS' POINT OF VIEW.

(Contributions to this Section are Invited)

### My Impression: The Unaided Composition of a Patient aged Eleven.

The patient's way of seeing things in the hospital may be interesting to others who have had the good fortune never to have been in one, and perhaps even to those whose duty it is to carry them on and to support them. I am therefore going to try to describe things as they appeared to me in my experience.

My own trouble was one of appendicitis as with many others who occupied beds in the same ward, and though the starving process with which my treatment commenced occupied most of my thoughts during the first few days after being admitted I soon began to take an interest in the doings and comings and goings of those about me. In due time my operation was successfully carried out, and yet another day or two of starving may be said to have completed the end of my suffering, and I was free to get well and strong again as quickly as possible.

When I was allowed to eat it may well be imagined that the food that came to my share was the most important event of the day, and though perhaps I felt a little inclined to grumble at the smallness of the portions given me it was no doubt best it should be so and now I have hinted at grumbling I had better finish it as far as the food was concerned. The eggs after having been collected from the patients, and gone through the cooking process, more or less successfully, were distributed according to marks, and it struck me that if a little more care had been taken to keep the shells whole there would probably have been a full egg to eat instead of sometimes that portion which had been unable to escape before they had been rescued from the water. In fact had they been fully cooked there would probably have been no egg left in the shell to eat, and this may account for the underdone condition we had them in. The butter on the bread could have been improved, and it was a relief to me that my visitors kindly brought me a pot of jam to kill its peculiar flavour. However, I never heard it called butter, perhaps it was not, and a welcome relief came when we had bread and dripping served.

Now another, and what appeared to me a rather unnecessary, game they used to play between five and six o'clock in the morning. I am speaking of November. This consisted of a general rousing out of sleep in order to have our beds made, washing ourselves or being washed, and a performance of other little matters which in the ordinary way could have been done quite as well at 7 A.M. Still, perhaps the night nurse did this to keep herself awake, so I do not complain even though sometimes a restless night spent in pain does not help one to look at it in this light at the time. By the way, speaking of rules, our visitors' hours are from 3 o'clock to 4.30 on Thursdays and Sundays, and the first Thursday after I was admitted the visitors were not allowed to come in until twenty minutes past the time because an Indian princess was expected to come on a tour of inspection and failed to keep appointment. She, however, came later, after the visitors had been admitted, and judging from the remarks made by my fellow patients, the impression was that they would sooner have seen their friends according to rule for the short time allowed, and it seemed hardly right that so many people had been kept waiting unnecessarily for one who had nobody there at all who was ill or who looked forward to her coming. I afterwards learned that the funds benefited when the visit was made, so this will excuse it.

And now about my neighbours. There was an old gentleman in the corner bed, of about seventy-five years of age, with white hair and a billy-goat beard, whom we nicknamed "Mouldy Mike" because of his peculiar ways. He was quite an original character, and though a little disgusting in some of his habits, was kindly disposed during his rather rare fits of sweet temper, and offered to show it by his gifts of bulls-eyes and other luxuries of which he was very fond.

Opposite to my bed lay a poor man who had already been there about four months in consequence of an accident while riding a motor bike, and expected to continue a patient for an indefinite time longer. He and I were very chummy, and between us we passed the time very pleasantly. My neighbour on the other side happened to be a boy about my own age, who had broken his arm while playing football, and with whom I shared my luxuries, and he his with me. Of the nurses and the ward sister I have nothing but praise to give, and considering the different ways and manners of the various patients they have under their charge they are most kind and considerate. For myself, had they forgotten the medicine instead of being so regular with it I should have been better pleased, but not perhaps better as soon.

Now I am well again and about I look back on my hospital experience with some amount of pleasure, and, though I am glad to be home again, I can understand the feeling which made one boy who was discharged during the time I was there cry for leaving the place; and, visiting the nurses myself since leaving, I was pleased to find that they were glad to see me, and I should not mind having to return there at any time if I became ill again.

